# Kinetic Study of the Thermal and Thermo-Oxidative Degradations of Polystyrene Reinforced with Multiple-Cages POSS

**DOI:** 10.3390/polym12112742

**Published:** 2020-11-19

**Authors:** Ignazio Blanco, Gianluca Cicala, Claudio Tosto, Francesco Agatino Bottino

**Affiliations:** 1 Department of Civil Engineering and Architecture, University of Catania an UdR-Catania Consorzio INSTM, Viale Andrea Doria 6, 95125 Catania, Italy; gcicala@unict.it (G.C.); claudio.tosto@unict.it (C.T.); 2 Department of Chemical Sciences, University of Catania, Viale Andrea Doria 6, 95125 Catania, Italy; fbottino@dii.unict.it

**Keywords:** polyhedral oligomeric silsesquioxanes, POSS, composites, thermal stability, kinetics, multiple cages, activation energy

## Abstract

A comprehensive kinetics degradation study is carried out on novel multiple cages polyhedral oligomeric silsesquioxane (POSS)/polystyrene (PS) composites at 5% (*w*/*w*) of POSS to assess their thermal behavior with respect to the control PS and other similar POSS/PS systems studied in the past. The composites are synthesized by in situ polymerization of styrene in the presence of POSSs and characterized by ^1^H-NMR. The characteristics of thermal parameters are determined using kinetics literature methods, such as those developed by Kissinger and Flynn, Wall, and Ozawa (FWO), and discussed and compared with each other and with those obtained in the past for similar POSS/PS composites. A good improvement in the thermal stability with respect to neat polymer is found, but not with respect to those obtained in the past for polystyrene reinforced with single- or double-POSS cages. This behavior is attributed to the greater steric hindrance of the three-cages POSS compared with those of single- or double-cage POSS molecules.

## 1. Introduction

Despite the pressing media campaign regarding the persistence of plastic in the environment, researchers are well aware that typical polymers are not indefinitely stable. Daily life is characterized by the constantly growing use of objects and devices made of plastic. Simple carbon-chain polymers, such as polyolefins [1,2], represent the widest sector of commercial polymer production due to the ease and affordability of manufacture by the processing of the molten polymer. With regards to the durability of these materials, we have to consider two different aspects: one related to their nature and the second related to their manufacturing. The chemical changes and/or degradation induced by processing may have a determining effect on the lifetime of the polymer in subsequent use. Polymers are typical organic molecules whose physical and mechanical properties depend on their long chains, rather than on special properties of the atoms and bonds composing these chains [3]. In particular, for thermoplastic polymers, this means that molecular rearrangements can start before the expected lifetime because they are typically more sensitive to both temperature and time than other materials. Their properties may change with time or with little temperature variations, even without any chemical reaction; thus, during both usage and storage, the polymers are subjected to a wide range of degradative influences, both physical and chemical [4,5,6]. Polyolefins are especially vulnerable to oxidation, becoming weakened and useless in a very short time; thus, most are unusable without additives that inhibit degradation chemistry [3]. It is therefore clear that polymer instability caused by weathering may be reduced by the different reactions contributing to this instability, rearrangements of the chemical structure, formation of oxidation products, crosslinking, and chain scission [7]. In the last three decades, thermal analysis has become an essential tool for the design and manufacturing of a polymeric material capable of satisfying the needs for which it was conceived. Purely thermal degradation is difficult to study, but if correctly carried out, it obtains useful information [8,9,10,11] not only for polymers but also for materials in general [12,13,14]. In particular, academic research has been devoted to enhancing the physical properties of polymers by adding low-molecular-weight substances such as plasticizers, stabilizers, and anti-blocking agents [15,16] or to modifying their structures and then verifying the effects of these modifications over the lifetime of the material under artificial accelerated conditions [17,18,19]. In this context, our research group at the University of Catania, in collaboration with eminent researchers in the field, tested the stabilization of different polymers, synthetics or naturals, such as polyethersulfone (PES), ethylene propylene diene monomer (EPDM), polyethylene oxide (PEO), and chitosan by the incorporation of polyhedral oligomeric silsesquioxanes (POSS) molecules [20,21,22,23,24]. The use of POSS in the making of polymer composites has grown exponentially in the last 30 years [25,26,27]. Represented in their most common form by the symbol T_8_ and having a diameter usually falling in the range of 1.5–3 nm, these molecules comprise a silicon and oxygen cage completed by organic groups that are covalently bonded with silicon atoms [28]. The ability to be dispersed at the molecular level and to play an active role in the reinforcement of polymeric materials, unlike other fillers such as organoclays [29], carbon nanotubes [30], and nanofibers [31,32], makes POSS unique among nano-reinforcements [33]. Particular focus was devoted to polystyrene (PS)/POSS composites by synthesizing PS first reinforced with single-cage POSS molecules [34,35] and then with double-cage POSS [36].

Composites of PS and multiple-cage POSS were synthesized by in situ polymerization of styrene in the presence of 5% molecular filler, and the following compounds were obtained:

[(C_4_H_9_)_7_Si_8_O_12_-O]_3_-Si-ArCH_3_/PS

[(C_5_H_9_)_7_Si_8_O_12_-O]_3_-Si-ArCH_3_/PS

After a spectroscopic investigation aiming to verify the presence of POSS in the synthesized composites, the composites’ thermal behavior in terms of resistance to thermal degradation along with the rate of degradation were evaluated by means of thermogravimetric analysis (TGA) and kinetics literature methods [37,38,39]. The calculated parameters, namely temperature at 5% mass loss (*T*_5%_) and apparent activation energy (*E_a_*) of degradation, were compared with each other and with those of the control PS to evaluate the differences in thermal behavior, if present.

## 2. Experimental

### 2.1. Materials

Tetrahydrofuran (THF), toluene, styrene, 2,2-azobis (isobutyronitrile) (AIBN), trichloroisobutyllsilane, trichlorocyclopentylsilane, p-tolyltrichlorosilane, and methanol were acquired from Aldrich Co. (St. Gallen, Switzerland). The latter four chemicals were used as received, whereas the other ones were treated as follows prior to use: THF was distilled over a Na-benzophenone mixture); toluene was stirred over calcium hydride for a day and then distilled in a nitrogen flow; styrene was purified in an inhibitor removal column; AIBN was crystallised from dry ethanol in a dark setting at room temperature. Cyclopentyl trisilanol POSS was prepared in agreement with literature reports [40,41], whereas trisilanol isobutyl POSS was acquired from Hybrid Plastics co. (Hattiesburg, MS, USA) and used as received.

**Figure 1:**
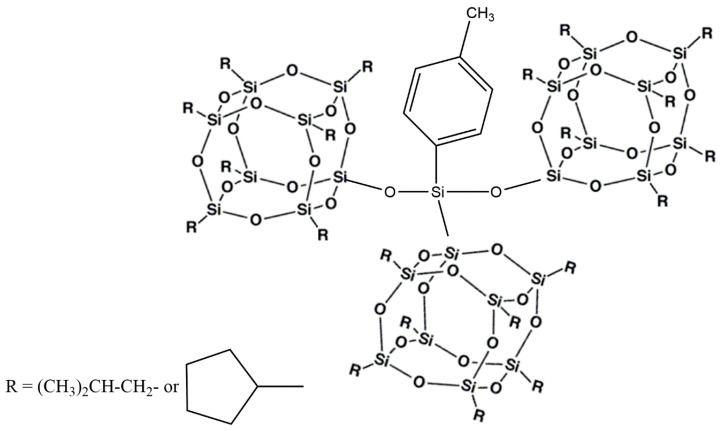
Molecular structure of the multiple polyhedral oligomeric silsesquioxanes (POSS) cages.

The synthesis procedure of 4-methyl phenyl (trioxyisobutyl POSS) silane and 4-methyl phenyl (trioxycyclopentyl POSS) silane together with their ^1^H NMR characterization are reported in our previous study [42] and the molecular structure of the obtained multiple-cage POSS is reported in Figure 1.

Samples **1** and **2** were obtained by in situ polymerization mixing in toluene 5% (*w*/*w*) of 4-methyl phenyl (trioxyisobutyl POSS) silane and 4-methyl phenyl (trioxycyclopentyl POSS) silane, respectively, with styrene. After the dissolution of each POSS and styrene monomer in toluene, the AIBN radical initiator was added to the mixture before being frozen in a liquid nitrogen bath, degassed with a vacuum pump, and then thawed. This operation was repeated three times before sealing under vacuum and heating, under stirring, at 343 K for a day. After this time, the obtained solution was precipitated by adding methanol and collected by filtration. The compounds were dried under vacuum at 313 K obtaining a yield of 76.6% and 83.2%, respectively, for samples **1** and **2**.

### 2.2. ^1^H-NMR Spectroscopy

^1^H-NMR characterization was performed in a Unity Inova instrument ^1^H 500 MHz (Varian, Palo Alto, CA, USA) by using deuterochloroform (CDCl_3_) as a solvent and tetramethylsilane (TMS) as an internal standard.

### 2.3. Thermogravimetric Analysis (TGA)

TGA was carried out with DTG-60 equipment (Shimadzu, Kyoto, Japan). Before performing measurements, the TGA apparatus was calibrated in agreement with a consolidate procedure reported in the literature [43,44] using, as standard materials, indium, tin, and zinc coded NIST SRM 2232, SRM 2220, and SRM 2221a, respectively. Samples of about 6 × 10^−3^ g were placed in an alumina open pan and degraded at eight different heating rates (*Φ* = 2, 5, 7.5, 10, 12.5, 15, 17.5, and 20 K∙min^−1^), in the temperature range of 298–973 K. Thermal degradations were performed in flowing nitrogen (0.02 L∙min^−1^), whereas thermos-oxidative degradations were performed in a static air atmosphere. TGA-obtained data were used to plot the percentage of an undegraded sample, (1 − *D*)%, as a function of temperature, where *D* is equal to:

$$D=\;\frac{\left(W_{0}-W\right)}{W_{0}}$$

where *W*_0_ and *W* are the masses at the starting point and during the TGA experiment, respectively. The derivative of the TG (DTG) curves were used to evaluate the temperature at the maximum degradation rate (*T_m_*), which was then used for kinetics calculation. All the considered *T* values were averaged over three runs, the maximum difference between the average and the experimental values being within ±1 K.

### 2.4. Fourier Transform Infrared Spectroscopy (FTIR)

The residues derived from TGA were analyzed by FTIR with a Spectrum 100 spectrometer (Perkin Elmer, Waltham, MA, USA) directly, without any pre-treatment, using a universal attenuated total reflection (ATR) sampling accessory. The spectra were collected at room temperature from 4000 to 650 cm^−1^ with a resolution of 4.0 cm^−1^.

## 3. Results and Discussion

During the in situ polymerization, we observed a slight increase in the POSS content in the obtained composites with respect to the initial mixture ratio (5% *w*/*w*) due to the formation of methanol soluble oligomers of PS. For this reason, before subjecting our samples to thermal characterization, we performed ^1^H-NMR measurements to verify the exact POSS content in the prepared PS composites. POSS content was calculated at 6.2% and 7.5% for compounds **1** and **2**, respectively, considering the ratio between the POSS hydrogen atoms and the PS hydrogen atoms.

**Figure 2:**
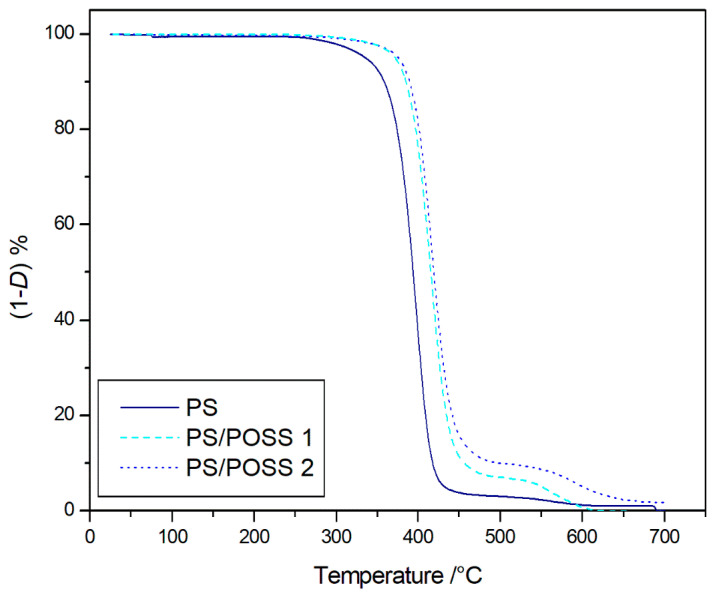
Thermogravimetric (TG) degradation curves, at 10 K∙min^−1^, in flowing nitrogen of samples **1** and **2** and control polystyrene (PS).

**Figure 3:**
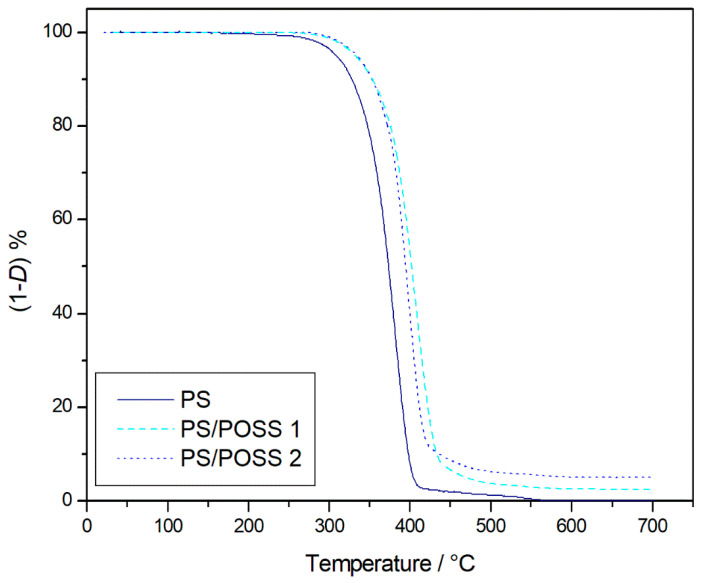
Thermogravimetric (TG) degradation curves, at 10 K·min^−1^, in the static air atmosphere of samples **1** and **2** and control PS.

Resistance to thermal degradation was first evaluated by comparing the TGA degradation curves at 10 K·min^−1^ from samples **1** and **2** with those of the control PS in inert and oxidative atmospheres, as shown in Figure 2 and Figure 3, respectively. In both investigated environments, an important shift in the beginning of composite degradation toward higher temperatures with respect to those of neat polymer appeared evident, at least qualitatively. The shift, highlighted by the TGA thermograms, was quantified thanks to the temperature at 5% mass loss, that is, 607.8 and 614.4 K in the air and 645.5 and 648.8 K in the nitrogen, for samples **1** and **2**, respectively. Given the *T*_5%_ values of 582.3 and 614.2 K found in the virgin PS in air and nitrogen, respectively, we recorded an increase ranging from 25 to 32 K in an oxidative environment and from 30 to 35 K in an inert one.

As *T*_5%_ revealed no significant differences among the investigated environments, TGA thermograms showed a different degradation that evolved in a single stage (653–723 K) in oxidative conditions (Figure 3), showing a contrasting additional stage of degradation (793–873 K) in an inert atmosphere (Figure 3). A solid residue was obtained at the end of the TGAs that was higher in oxidative (Figure 3) than in inert atmosphere (Figure 2), which was analyzed by FTIR analysis and associated with the presence of SiO_2_, whereas no band for un-decomposed PS or its decomposition products were detected (Figure SM in the Supplementary Materials).

The least square treatment of the data reported in Table 1 and Table 2 was performed by using Kissinger [37] and Flynn, Wall, and Ozawa (FWO) [38,39] equations to calculate the apparent activation energy of degradation (*E_a_*).

The Kissinger Equation,

(1) $$\ln\left(\frac{\Phi }{T_{m}^{2}}\right)=\ln\left(\frac{nRAW_{m}^{n-1}}{E_{a}}\right)-\frac{E_{a}}{RT_{m}},$$

allows the calculation of *E_a_* values through the straight lines obtained reporting ln(*Φ*/*T_m_*^2^) as a function of 1/*T_m_* at various heating rates. In Equation (1), *Φ* is the heating rate, *T_m_* is the temperature at maximum rate of weight loss, *n* is the apparent reaction order, *R* is the universal gas constant, *A* is the pre-exponential factor, and *W_m_* is the weight of the sample at the maximum rate of mass loss. The Kissinger equation yields reliable *E_a_* values only when conversion does not practically vary the heating rate, and it should not be considered an isoconversional method [45,46,47]. Thus, to obtain a reliable estimation of the kinetics of degradation of the prepared compounds, we checked that the degree of conversion did not vary with heating rate. In addition, the obtained TGA data at the various heating rates were treated through the FWO integral isoconversional method based on the following Equation:

(2) $$\ln\Phi \;=ln\left(\frac{AE_{\alpha }}{g\left(\alpha \right)R}\right)-5.3305-1.052\frac{E_{\alpha }}{RT_{\alpha }}$$

where *T_α_* is the temperature when, at a fixed heating rate *Φ*, a certain conversion *α* is achieved.

Degradation *E_a_* values obtained by the Kissinger equation, together with their corresponding regression coefficients, are reported in Table 3 and Table 4 for oxidative and inert environments, respectively.

**Figure 4:**
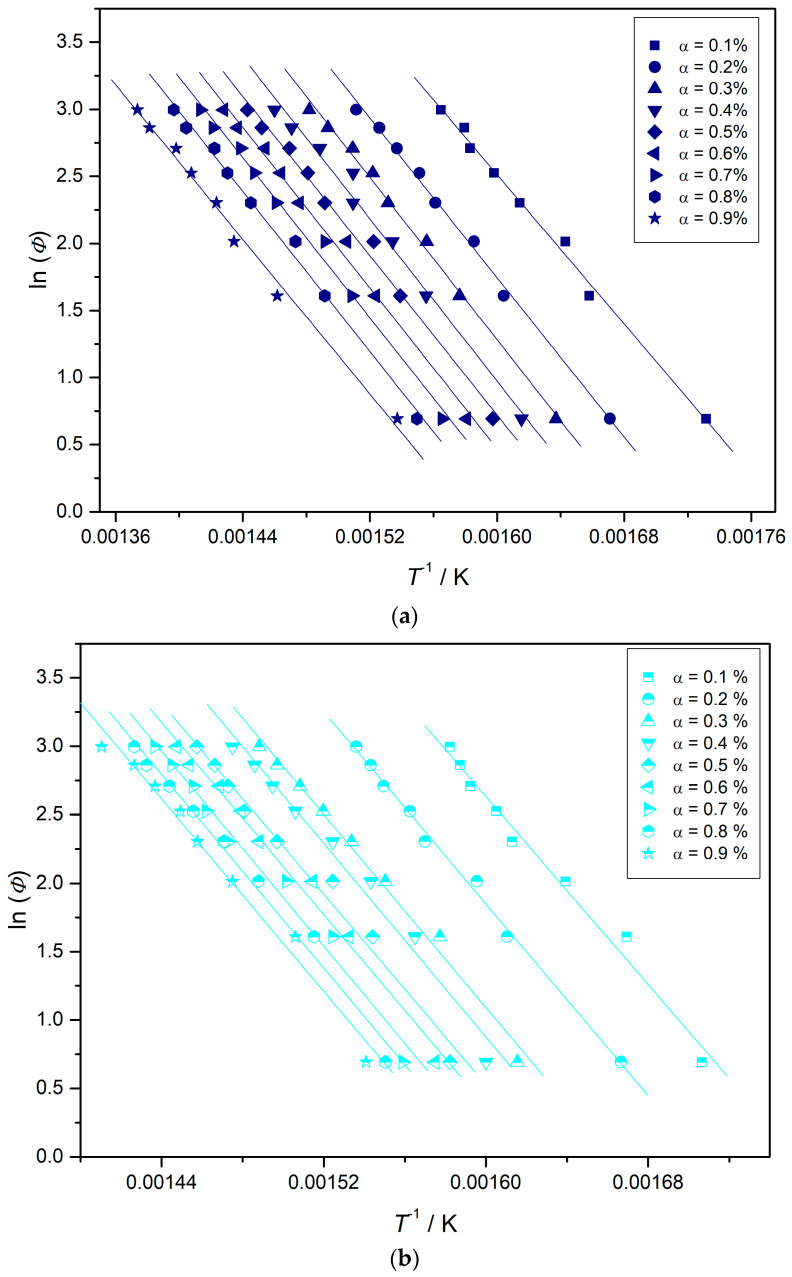

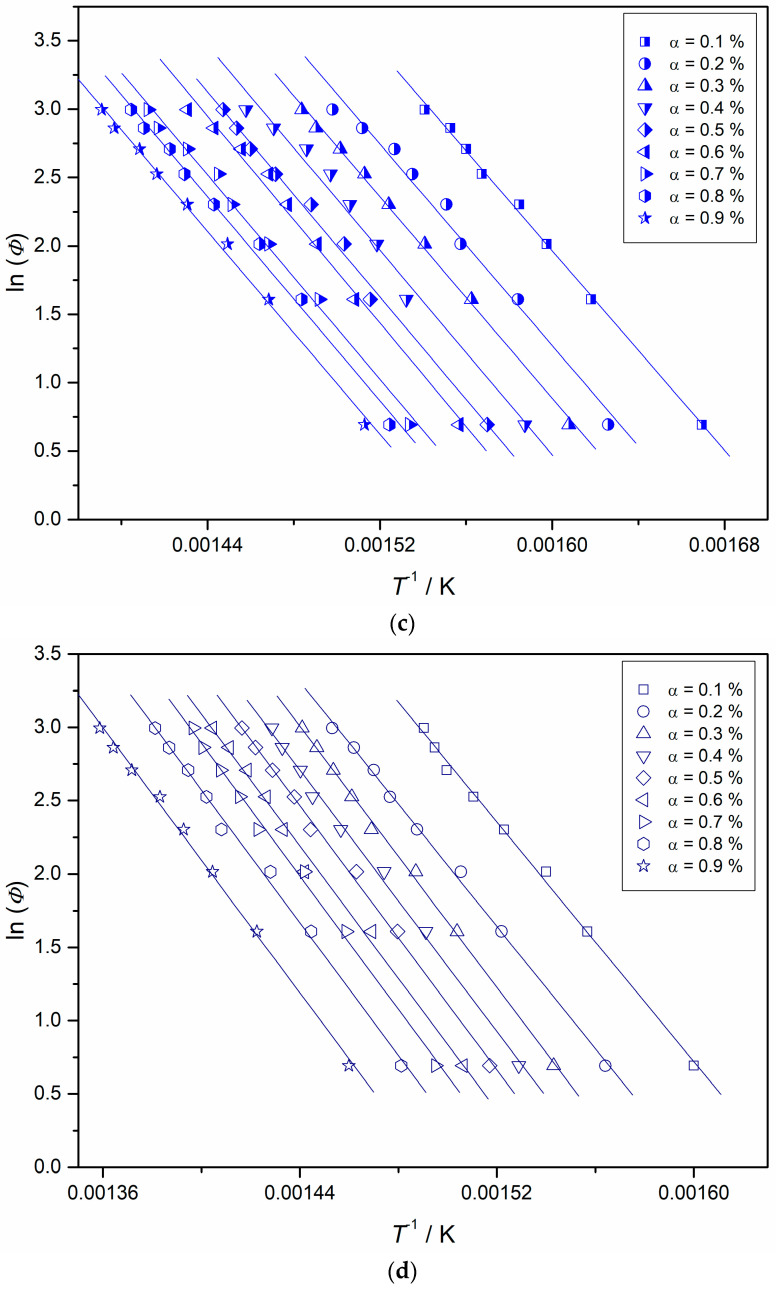

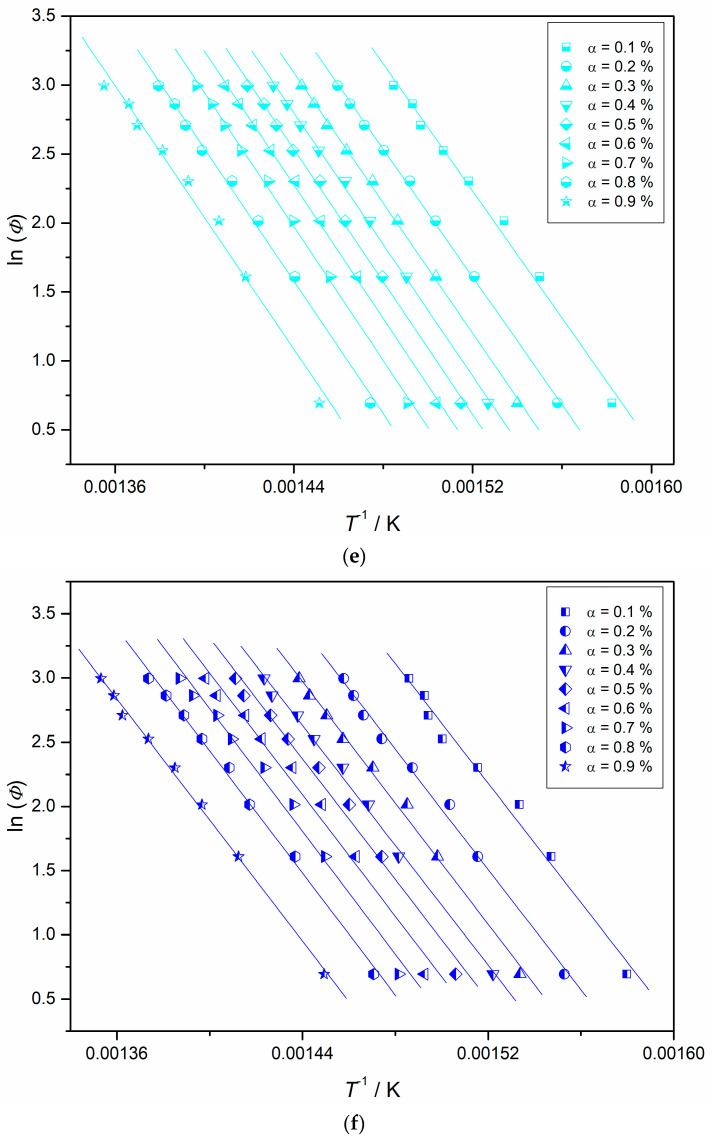
Flynn, Wall, and Ozawa straight lines for PS (**a**), sample 1 (**b**), sample 2 (**c**) in flowing nitrogen and PS (**d**); sample **1** (**e**) and sample **2** (**f**) in static air atmosphere at various degrees of conversion (α).

Activation energy values at a fixed value of conversion (*E*_α_) were obtained by the FWO equation through the ln(*Φ*) vs. 1/*T_α_* plots reported in Figure 4a–f at the different heating rates considered, assuming that the Doyle’s approximation is valid for all degrees of conversion [48].

As shown in Table 5, a very good agreement among the *E_a_* values obtained through Equations (1) and (2) was found, showing a large enhancement for the composites reinforced with the multiple-cage POSS in static air atmosphere, amounting approximately to 20–25 kJ·mol^−1^. Conversely, in an inert environment, the average (among the Kissinger and FWO methods) degradation *E_a_* values showed a lesser increase with respect to the value of the control PS, about 10–15 kJ·mol^−1^. Kinetics data confirmed the key role of multiple-cage POSS in the thermo-oxidative degradation process of the obtained PS composites that let down the degradation rate, and, as demonstrated by the *T*_5%_ values, enhanced the overall thermal stability of the composites. In an inert environment, the role of POSS seems to be to mitigate, even if, in any case, there is a slight slowdown of the degradation kinetics (Table 5).

When we looked at our previous research regarding the kinetics of the degradation of similar PS/POSS composites but with single- [49,50,51] or double-cage structures [52,53], we observed that the slowdown in degradation kinetics for the triple-cage POSS-reinforced polystyrene was much less pronounced, about 20–30 kJ∙mol^−1^ lower than in the systems studied in the past. This behavior could be attributed to the difficulty of obtaining multiple-cage POSS, which are sterically bulkier than single-cage POSS, to disperse themselves in the polymer matrix. In addition, the same steric hindrance can likely lead to a reduced level of interaction with the matrix, which can be translated into a reduced reinforcement action.

## 4. Conclusions

The design and preparation of new PS composites reinforced with triple-cage POSS, uniquely functionalized with isobutyl and cyclopentyl groups, were carried out to verify if the presence of a higher number of POSS cages in the same molecule, dispersed in the matrix, leads to a further increase of material stabilization.

The kinetics study of the thermal and thermo-oxidative degradations of the prepared composites and the control polymer showed an increase in the thermal stabilization of the obtained materials. However, this was not comparable with the results obtained in the past for similar systems obtained by adding molecules with one or at least two POSS cages to the matrix.

We attribute this result to the increase in the steric hindrance of the multiple-cage POSS that leads to dispersion difficulty at the molecular level in the polymer matrix, reducing the level of interaction and thus the reinforcement action.

## Figures and Tables

**Table 1:** Temperatures at maximum rate of weight loss (*T_m_*) for the degradation of PS and synthesized POSS/PS nanocomposites in static air atmosphere.

	PS	Sample 1	Sample 2
*Φ* (K·min^−1^)	*T_m_* (K)	*T_m_* (K)	*T_m_* (K)
2	640	643	651
5	654	658	667
7.5	668	666	673
10	679	678	680
12.5	684	681	687
15	689	684	693
17.5	694	695	700
20	699	696	704

**Table 2:** Temperatures at maximum rate of weight loss (*T_m_*) for the degradation of PS and synthesized POSS/PS nanocomposites in flowing nitrogen.

	PS	Sample 1	Sample 2
*Φ* /(K·min^−1^)	*T_m_*/K	*T_m_*/K	*T_m_*/K
2	664	664	664
5	678	677	679
7.5	686	684	685
10	696	691	693
12.5	698	696	697
15	702	699	701
17.5	705	703	705
20	710	706	707

**Table 3:** Regression coefficients and apparent activation energies (*E_a_*) of degradation by the Kissinger equation for PS and synthesized POSS/PS nanocomposites in static air.

Sample	a ^(a)^	b·10^−3^ (K) ^(b)^	r ^(c)^	*E_a_* (kJ·mol^−1^)
PS	11.7 (±1.4)	15.2 (±0.9)	0.9890	126 (±7)
1	14.6 (±1.8)	17.1 (±1.2)	0.9844	142 (±10)
2	15.7 (±2.0)	18.1 (±1.4)	0.9831	150 (±12)

^(a)^ a = ln(nRAW_m_^n−1/^*E_a_*); ^(b)^ b = *E_a_*/R; ^(c)^ product moment correlation coefficient.

**Table 4:** Regression coefficients and apparent activation energies (*E_a_*) of degradation by the Kissinger equation for PS and synthesized POSS/PS nanocomposites in flowing nitrogen.

Sample	a ^(a)^	b·10^−3^ (K) ^(b)^	r ^(c)^	*E_a_* (kJ·mol^−1^)
PS	20.8 (±1.4)	21.9 (±1.0)	0.9937	182 (±8)
1	22.6 (±1.1)	23.2 (±0.7)	0.9968	193 (±6)
2	23.5 (±1.4)	23.7 (±1.0)	0.9951	197 (±8)

^(a)^ a = ln(nRAW_m_^n−1/^*E_a_*); ^(b)^ b = *E_a_*/R; ^(c)^ product moment correlation coefficient.

**Table 5:** Apparent activation energies (*E_a_*) of degradation for PS and synthesized POSS/PS nanocomposites obtained by the Kissinger and Flynn–Wall–Ozawa (FWO) equation in static air atmosphere and in flowing nitrogen.

Compounds	Static Air		Nitrogen Flow	
	Kissinger	FWO	Kissinger	FWO
	*E_a_* (kJ·mol^−1^)	*E_a_* (kJ·mol^−1^)	*E_a_* (kJ·mol^−1^)	*E_a_* (kJ·mol^−1^)
**PS**	126 (±7)	123 (±4)	182 (±8)	183 (±4)
**1**	142 (±7)	146 (±7)	193 (±6)	197 (±4)
**2**	150 (±12)	154 (±5)	197 (±8)	196 (±6)

## Supplementary Materials

The following are available online at https://www.mdpi.com/2073-4360/12/11/2742/s1, Figure SM1: FTIR spectra of the solid residues at 700 °C for the samples 1 and 2, Table SM1: Regression coefficients and apparent activation energies (*E*_a_) of degradation by the FWO integral isoconversional method for PS in static air, Table SM2: Regression coefficients and apparent activation energies (*E*_a_) of degradation by the FWO integral isoconversional method for PS in flowing nitrogen, Table SM3: Regression coefficients and apparent activation energies (*E*_a_) of degradation by the FWO integral isoconversional method for sample 1 in static air, Table SM4: Regression coefficients and apparent activation energies (*E*_a_) of degradation by the FWO integral isoconversional method for sample 1 in flowing nitrogen, Table SM5: Regression coefficients and apparent activation energies (*E*_a_) of degradation by the FWO integral isoconversional method for sample 2 in static air, Table SM6: Regression coefficients and apparent activation energies (*E*_a_) of degradation by the FWO integral isoconversional method for sample 2 in flowing nitrogen.
