# Supplementary material for: Kinetic Study of the Thermal and Thermo-Oxidative Degradations of Polystyrene Reinforced with Multiple-Cages POSS
Source: Polymers (Basel). 2020 Nov 19;12(11):2742. doi: 10.3390/polym12112742 (PMC7699152; doi:10.3390/polym12112742)
Supplement: Supplementary file 1 [file polymers-12-02742-s001.pdf]

H NMR data

isobutyl POSS chloride $[(C_4H_9)_7 Si_8O_{12}]$–Cl
$_1$H NMR: 1.87 (m, 7H), 0.95 (m, 42H), 0.62 (m, 14H).
Analysis Calculated for $C_{28}H_{63}ClO_{12}Si_8$: C 39.47, H 7.45.
Found: C 39.02, H 7.58.

cyclopentyl POSS chloride $[(c–C_5H_9)_7Si_8O_{12}]$–Cl
$_1$H NMR: 1.77 (m, 14H), 1.54 (m, 42H), 1.04 (m, 7H).
Analysis Calculated for $C_{35}H_{63}ClO_{12}Si_8$: C 44.91, H 6.79.
Found: C 44.37, H 6.88.

isobutyl POSS mono-ol $[(C_4H_9)_7 Si_8O_{12}]$–OH
$_1$H NMR: 1.85 (m, 7H), 0.96 (m, 42H), 0.56 (m, 14H).
Analysis Calculated for $C_{28}H_{64}O_{13}Si_8$: C 40.35, H 7.74.
Found: C 39.96, H 7.68.

Cyclopentyl POSS mono-ol $[(c–C_5H_9)_7Si_8O_{12}]$–OH
$_1$H NMR: 1.74 (m, 14H), 1.56 (m, 42H), 0.98 (m, 7H).
Analysis Calculated for $C_{35}H_{64}O_{13}Si_8$: C 45.84, H 7.03.
Found: C 44.16, H 7.10.

4-methyl phenyl (trioxyisobutyl POSS) silane $[(C_4\text{-}H_9)_7Si_8O_{12}–O]_3$–Si–Ar$CH_3$
$_1$H NMR: 7.60 (dd, 2H), 7.15 (dd, 2H), 2.38 (s, 3H),
1.81 (m, 21H), 0.94 (m, 126H), 0.59 (m, 42H). Analysis
Calculated for $C_{91}H_{196}O_{39}Si_{25}$: C 41.94, H 7.54. Found: C
41.17, H 7.68.

4-methyl phenyl (trioxycyclopentyl POSS) silane $[(C_5H_9)_7Si_8O_{12}–O]_3$–Si–Ar$CH_3$
$_1$H NMR: 7.63 (dd, 2H), 7.12 (dd, 2H), 2.35 (s, 3H),
1.73 (m, 42H), 1.54 (m, 126H), 0.99 (m, 21H). Analysis
Calculated for $C_{112}H_{196}O_{39}Si_{25}$: C 47.06, H 7.54. Found: C
41.17, H 6.87.

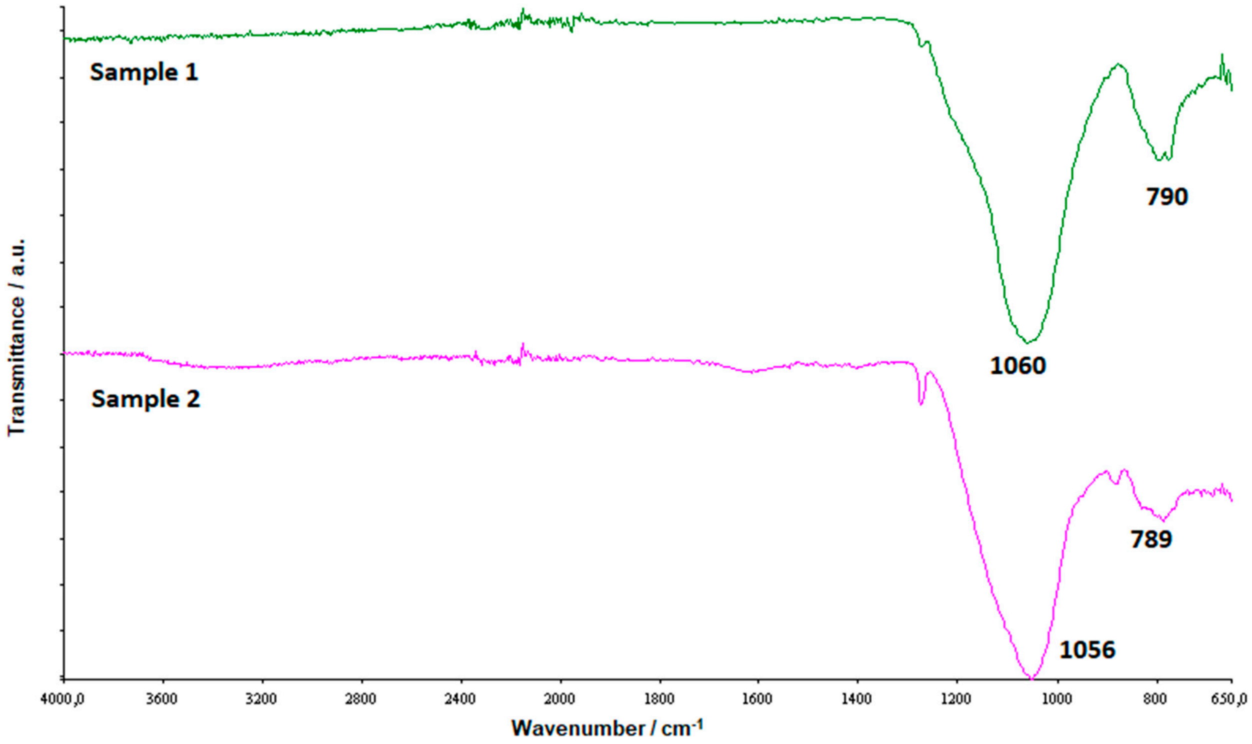

Figure SM1. FTIR spectra of the solid residues at 700 °C for the samples **1** and **2**.

Table SM1. Regression coefficients and apparent activation energies (E_a) of degradation by the FWO integral isoconversional method for **PS** in static air.

α	*a*	$b \cdot 10^{-3}$ /K	r	E_a /(kJ·mol^{-1})
0.1	24.8 (± 0.7)	13.9 (± 0.4)	0.9968	116 (± 3)
0.2	25.5 (± 0.6)	14.8 (± 0.4)	0.9979	123 (± 3)
0.3	25.4 (± 0.6)	15.1 (± 0.4)	0.9977	125 (± 3)
0.4	25.1 (± 0.6)	15.1 (± 0.7)	0.9931	125 (± 6)
0.5	24.5 (± 0.8)	14.8 (± 0.6)	0.9956	123 (± 5)
0.6	24.4 (± 0.8)	15.0 (± 0.5)	0.9964	125 (± 4)
0.7	24.2 (± 0.8)	14.9 (± 0.6)	0.9957	124 (± 5)
0.8	23.9 (± 0.7)	14.9 (± 0.5)	0.9967	124 (± 4)
0.9	22.8 (± 0.7)	14.4 (± 0.5)	0.9960	120 (± 4)

Table SM2. Regression coefficients and apparent activation energies (E_a) of degradation by the FWO integral isoconversional method for **PS** in flowing nitrogen.

α	*a*	$b \cdot 10^{-3}$ /K	r	E_a /(kJ·mol^{-1})
0.1	33.4 (± 0.7)	20.4 (± 0.4)	0.9986	170 (± 3)
0.2	33.3 (± 0.6)	20.8 (± 0.4)	0.9989	173 (± 3)
0.3	35.1 (± 0.6)	22.2 (± 0.4)	0.9990	185 (± 3)
0.4	34.8 (± 0.8)	22.3 (± 0.5)	0.9982	185 (± 4)
0.5	34.8 (± 0.7)	22.5 (± 0.5)	0.9986	187 (± 4)
0.6	34.6 (± 1.1)	22.5 (± 0.8)	0.9966	187 (± 7)
0.7	34.7 (± 0.8)	22.7 (± 0.6)	0.9981	189 (± 5)
0.8	34.2 (± 0.8)	22.6 (± 0.6)	0.9979	188 (± 5)
0.9	33.7 (± 0.7)	22.6 (± 0.5)	0.9986	188 (± 4)

Table SM3. Regression coefficients and apparent activation energies (E_a) of degradation by the FWO integral isoconversional method for sample **1** in static air.

α	a	$b \cdot 10^{-3}$ /K	r	E_a /(kJ·mol^{-1})
0.1	30.1 (± 1.4)	17.2 (± 0.9)	0.9923	143 (± 7)
0.2	29.9 (± 0.8)	17.5 (± 0.5)	0.9977	145 (± 4)
0.3	29.3 (± 1.1)	17.6 (± 0.7)	0.9950	146 (± 6)
0.4	29.1 (± 1.6)	17.7 (± 1.0)	0.9898	147 (± 8)
0.5	28.4 (± 1.4)	17.4 (± 0.9)	0.9915	145 (± 7)
0.6	28.4 (± 1.2)	17.5 (± 0.8)	0.9939	145 (± 7)
0.7	28.6 (± 1.5)	17.8 (± 1.0)	0.9907	148 (± 8)
0.8	28.3 (± 1.2)	17.7 (± 0.8)	0.9932	147 (± 7)
0.9	27.9 (± 1.4)	17.6 (± 1.0)	0.9911	146 (± 8)

Table SM4. Regression coefficients and apparent activation energies (E_a) of degradation by the FWO integral isoconversional method for sample **1** in flowing nitrogen.

α	a	$b \cdot 10^{-3}$ /K	r	E_a /(kJ·mol^{-1})
0.1	37.2 (± 1.3)	23.0 (± 0.9)	0.9958	191 (± 7)
0.2	36.8 (± 0.5)	23.2 (± 0.3)	0.9994	193 (± 2)
0.3	37.1 (± 0.5)	23.6 (± 0.4)	0.9993	196 (± 3)
0.4	37.1 (± 0.5)	23.8 (± 0.4)	0.9993	198 (± 3)
0.5	37.1 (± 0.6)	24.0 (± 0.4)	0.9990	199 (± 3)
0.6	37.1 (± 0.5)	24.2 (± 0.3)	0.9994	201 (± 2)
0.7	36.9 (± 0.7)	24.2 (± 0.5)	0.9989	201 (± 4)
0.8	36.1 (± 0.9)	24.0 (± 0.6)	0.9980	199 (± 5)
0.9	35.5 (± 1.6)	23.9 (± 1.2)	0.9930	199 (± 10)

Table SM5. Regression coefficients and apparent activation energies (E_a) of degradation by the FWO integral isoconversional method for sample **2** in static air.

α	a	$b \cdot 10^{-3}$ /K	r	E_a /(kJ·mol^{-1})

0.1	31.1 (± 0.6)	18.2 (± 0.3)	0.9989	151 (± 2)
0.2	30.7 (± 1.5)	18.4 (± 0.9)	0.9918	153 (± 7)
0.3	30.7 (± 0.6)	18.4 (± 0.4)	0.9988	153 (± 3)
0.4	30.4 (± 1.5)	18.7 (± 1.0)	0.9915	155 (± 8)
0.5	30.1 (± 0.9)	18.7 (± 0.6)	0.9970	155 (± 5)
0.6	30.2 (± 1.2)	18.9 (± 0.8)	0.9948	157 (± 7)
0.7	29.4 (± 0.9)	18.7 (± 0.6)	0.9965	155 (± 5)
0.8	29.2 (± 0.9)	18.6 (± 0.6)	0.9965	155 (± 5)
0.9	28.8 (± 0.7)	18.5 (± 0.5)	0.9981	154 (± 4)

Table SM6. Regression coefficients and apparent activation energies (E_a) of degradation by the FWO integral isoconversional method for sample **2** in flowing nitrogen.

α	*a*	$b \cdot 10^{-3}$ /K	r	E_a /(kJ·mol^{-1})
0.1	37.5 (± 1.7)	23.3 (± 1.1)	0.9932	194 (± 9)
0.2	37.1 (± 1.1)	23.4 (± 0.7)	0.9970	194 (± 6)
0.3	36.0 (± 1.0)	23.6 (± 0.7)	0.9974	196 (± 6)
0.4	36.2 (± 0.9)	23.3 (± 0.6)	0.9977	194 (± 5)
0.5	36.2 (± 1.6)	23.5 (± 1.1)	0.9933	195 (± 9)
0.6	36.1 (± 1.9)	23.6 (± 1.3)	0.9909	196 (± 11)
0.7	36.2 (± 1.6)	23.9 (± 1.1)	0.9937	199 (± 9)
0.8	35.7 (± 0.9)	23.8 (± 0.6)	0.9980	198 (± 5)
0.9	34.9 (± 0.7)	23.6 (± 0.5)	0.9985	196 (± 4)
